# Fungal community characteristics and driving factors during the decaying process of *Salix psammophila* sand barriers in the desert

**DOI:** 10.1371/journal.pone.0258159

**Published:** 2021-10-01

**Authors:** Yumei Liang, Yong Gao, Ruidong Wang, Xia Yang

**Affiliations:** Key Laboratory of State Forest Administration for Desert Ecosystem Protection and Restoration, College of Desert Control Science and Engineering, Inner Mongolia Agricultural University, Hohhot, China; Feroze Gandhi Degree College, INDIA

## Abstract

Wood-inhabiting fungi are crucial to wood decay and decomposition in *S*. *psammophila* sand barriers, which in turn consumingly influence nutrient dynamics in desert soils. In the case of an extremely arid desert, as opposed to forests, little of known about the fungal community composition of decaying wood and the effects of decomposing wood on soil physical and chemical properties. Combined with high-throughput gene sequencing technology, we investigated the relationships between microenvironment factors with fungal community composition and diversity during the decomposition of *Salix psammophila* sand barriers. The results showed that the destruction of lignocellulose components during the decay process of *S*. *psammophila* sand barrier alters the physical and chemical properties of the surrounding soil. Compared with one-year sand barrier, lignin and cellulose of seven-year *S*. *psammophila* sand barrier decreased by 40.48% and 38.33%, respectively. Soil available potassium and available nitrogen increased by 39.80% and 99.46%, respectively. We confirmed that soil available nitrogen, soil pH and soil moisture content significantly affected the fungal community distribution of *S*. *psammophila* sand barriers. Sordariomycetes are mainly affected by the positive correlation of soil pH, while Eurotiomycetes are most affected by the positive correlation of soil moisture content and soil porosity. Although our results highlighted the importance of bidirectional interactions between fungi in decayed sand barriers and soil properties, their contribution to the desert ecosystem still needs further confirmation from future studies. However, overall our findings improved the current understanding of the sand barrier-soil interactions on the process of ecological restoration.

## 1. Introduction

The natural shrub of *Salix psammophila* is widely grown in the desert area of northern China [1]. It has strong drought resistance and high-temperature resistance and is the preferred sand-fixing afforestation species in wind-sand shelterbelt systems. *S*. *psammophila* branches can be used as a sand barrier material to control the speed and direction of wind-sand flow [2]. Compared to other materials such as clay and pebbles, *S*. *psammophila* checkerboard sand barrier has certain elasticity and air permeability. It is a typical mechanical protection measure in sandy areas. As a natural biological resource, it is a fabulous mechanical sand barrier material because of its low cost and no pollution, thus it is used in fixing mobile sand dunes as sand barriers [2,3]. The installation of mechanical sand barriers can avoid wind erosion caused by direct friction between airflow and the ground surface [4]. It can reduce transit wind speeds and increase surface roughness. In general, *S*. *psammophila* mechanical sand barriers can not only ensure the normal growth of vegetation but also achieve the effect of sand fixation [3]. Numerous studies have shown that *S*. *psammophila* sand barriers will change in the physical and chemical properties of the aeolian sandy soil after setting [5]. More specifically, it plays a role in increasing the fine particles and soil organic carbon in dune topsoil [6], which promotes the process of ecological restoration and is a kind of sustainable desertification control measure.

After the *S*. *psammophila* branches are cut and set in the dunes, the plant’s tissues or organs are removed and the cells stop growing or die. Exposed to the field environment for a long time, the sand barriers degrade due to natural factors such as weathering, biodegradation, alternated desorption-absorption, and microbial decomposition [7,8]. These biotic and abiotic processes combine to impact the degradation degree of *S*. *psammophila* sand barriers. However, wood decomposition rates essentially are driven by microbial communities [9], especially assemblages of saprophytic fungi [10,11]. Many fungi undergo a succession of taxonomic groups able to decompose biopolymers (cellulose, hemicellulose, lignin, and other aromatic compounds) [12,13]and use comprehensive enzymatic systems to decompose lignocellulose [14]. Within the first stage of the decomposition of natural biomaterials or woods, the fungal communities involved are mostly endophytes [13], while in the second stage, the sand barrier woods show different dominating taxa. Despite these numerous observational studies on the composition and diversity of wood-inhabiting fungal communities in forest ecosystems [15,16], the relative importance and interaction of the host and environment in extremely arid desert ecosystems remain unclear. There is very limited information on the changes in fungal community composition during the decomposition of *S*. *psammophila* sand barriers. This lack of knowledge hinders a deeper understanding of the diversity of wood-inhabiting fungal communities in extremely arid environments.

Among desert microbial communities, fungi are the most stress-resistant eukaryotes [17]. They are a vital component of desert ecosystems because they control the decay and decomposition of organic residues, dead roots, wood and litter [18]. Many studies have shown that differences in the wood decomposition of natural biological materials were driven by soil contact and associated with increased moisture content [16]. It has been confirmed that the stable sand-buried section of the sand barrier has been buried in the sand for a long time and its decay degree is the most serious than that of other parts (atmosphere-exposed section and atmosphere-sand dynamic interface) [8,19]. The aging of the sand-buried section of *S*. *psammophila* sand barriers further increases the water absorption capacity and promotes the growth and reproduction of wood-inhabiting fungi. Furthermore, due to the poor nutrient content in sandy soils, the decomposition process of *S*. *psammophila* sand barrier is regarded as a significant source of nutrients for desert soils [5,6]. Soil serves as a medium and is a major route to deadwood for wood-inhabiting fungi colonization [20]. Following wood colonization, fungal mycelium can grow rapidly in suitable environmental conditions and then they secrete the wood decomposition enzymes such as hydrolases and oxidoreductases to decompose the polymeric polysaccharides (cellulose, hemicellulose and lignin). Different fungal taxa differ greatly in their ability to decompose the biopolymers of the wood internal substrate, leading to variation in decomposition across fungal communities [21,22]. Hence, the community species composition and diversity of wood-inhabiting fungi in *S*. *psammophila* sand barrier directly affect the content of soil nutrients.

In a word, long-term exposure to a desert environment, wood decomposition of *S*. *psammophila* sand barriers were strongly associated with both fungal community composition and microenvironmental conditions. Specific microenvironmental of the stable sand-buried section of the barriers include two aspects. On the one hand, the internal substrate of decayed sand barriers serves as a resource to provide nutrients for the growth and reproduction of the fungus [23]. On the other hand, the external surrounding soil conditions directly affect the characteristics of sand barriers and the growth of fungi. However, we have little understanding about the complex interactions among wood-inhabiting fungi species composition of *S*. *psammophila* sand barriers, properties of the surrounding soil and chemical composition of decayed sand barriers or woody debris. This knowledge gap largely hinders our understanding of the wood-inhabiting fungi in desert areas.

In this study, we hypothesized that the chemical components of *S*. *psammophila* sand barriers would be degraded to some extent and improve the physical and chemical properties of the soil with the increase of ecological service years. We also hypothesized that the fungal community composition and relative abundance in *S*. *psammophila* sand barriers would change, along with the increase of decay, as well as the fungi in the major taxa being influenced by some specific environmental factors. To test these hypotheses, we studied in detail the main chemical constituents of the sand barriers and the physical and chemical properties of the surrounding soil, analyzed the composition of the fungal community in combination with high-throughput sequencing technology. We further evaluated the interrelationships between fungal community characteristics and microenvironmental factors of long-established *S*. *psammophila* sand barriers in deserts.

## 2. Materials and methods

### 2.1 Site description and sampling design

The study area is on both sides of the S24 Xingba Highway through the sand in the Kubqi Desert in Hangjin Banner, Ordos, Inner Mongolia (108°46′54″E, 40°26′43″N). To ensure the safe passage of vehicles, semi-concealed *S*. *psammophila* checkerboard sand barriers were used to fix the transiting drifting sand. The regional climate is characterized as a typical temperate continental monsoon climate, controlled by the westerly circulation aloft all year round and influenced by the monsoon circulation near the surface. There are sufficient sunshine and a dry climate, with an annual average temperature of 6.3°C, average sunshine hours of 3193 h, annual average wind speed of 4.4 m/s, and a frost-free period of 135 d. The landform types are mobile dunes, semi-mobile dunes and fixed dunes. The main vegetation types contain *Salix psammophila*, *Artemisia ordosica*, *Caragana korshinskii*, *Agriophyllum squarrosum* and *Corispermum hyssopifolium*. The subsurface part of the *S*. *psammophila* sand barriers, which has been buried in the sand for a long time, was badly decayed. Therefore, we focused on this section necessarily.

**Fig 1:**
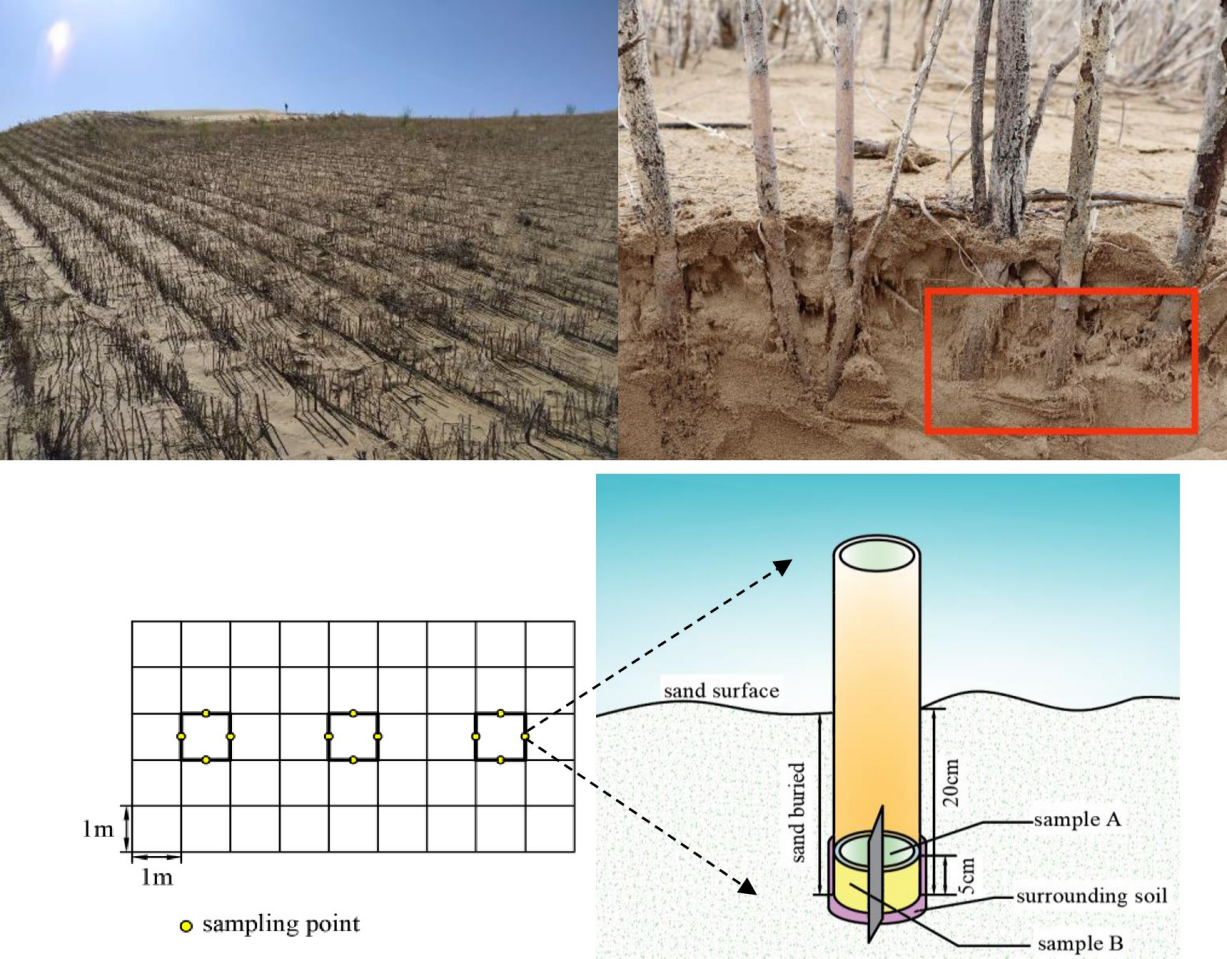
The schematic diagram for sample plots and sampling of *S*. *psammophila* sand barriers.

The experimental materials were collected in November 2019. Space instead of time approach was used by us to locate the test area, which represents the decay characteristics of *S*. *psammophila* sand barriers well. After determining the setting year of the sand barriers, the sample plots were divided accurately. We selected four sample plots of *S*. *psammophila* sand barriers with a specification of 1 m × 1 m that had been setting for one, three, five and seven years for investigation and sampling. We focused on three subplots, spaced 2 m apart, selected in the middle of each sample plot on the same contour (Fig 1). Samples of sand barriers with a diameter of 1.8 ± 0.02 cm and a sand burial depth of 20 cm were selected from the middle of each barrier side (east, west, south and north directions). The test materials were 5 cm samples from the bottom of the sand barriers. Each sample was divided into two equal sub-samples (A and B) and crushed respectively. Subsequently, the two sub-samples of four samples from the same subplot were mixed correspondingly separately as one sample. Then the mixture was transferred to sterile centrifuge tubes (CORNING CentriStar TM, USA). Sub-sample A was used for the extraction of fungal DNA from *S*. *psammophila* sand barriers, transported on dry ice to the laboratory and stored at −80°C. Sub-sample B was used for the determination of cellulose, hemicellulose and lignin content of sand barriers. All the tools used in the sampling process are sterilized.

As a primary study on general sand barriers decomposition, we considered surrounding adhering soil, which was influenced by the decaying sand barriers. We obtained the soil that was shaken off the sand barrier’s surface. The sandy soil was combined, homogenized and sieved through a 2 mm mesh to eliminate gravel and litter. The surrounding adhering soil samples were used to determine soil physicochemical properties, which were transferred to the laboratory in polyethylene bags, and air-dried in a ventilated place.

### 2.2 Soil physicochemical properties

Soil physicochemical properties including soil moisture content, organic matter, porosity, soil available nutrients such as phosphorus, potassium, and nitrogen were analyzed at the Key Laboratory of State Forest Administration for Desert Ecosystem Protection and Restoration, Inner Mongolia Agricultural University, China.

The soil moisture content (MC) was determined gravimetrically after oven drying at 105°C. The soil organic matter (OM) was analyzed by potassium dichromate oxidation colorimetry [24]. The absorbance of the supernatant was measured at a wavelength of 590 nm. The soil organic matter (SOM) was analyzed by Bao method [25]. The pH meter was calibrated (PHS-3C, Lexi, Shanghai, China), and soil pH was measured a 1:5 soil/water suspension after shaking thoroughly for 10 min at 25°C [26]. Soil porosity (SP) was determined using the ring cutting method. The content of available potassium (AK) in soil was obtained by NH4OAc extraction and flame photometry [27]. The soil available phosphorus content (AP) was obtained by NaHCO_3_ extraction and molybdenum vanadate colorimetric method [28]. Soil available nitrogen (AN) was determined by indophenol blue colorimetric method [25].

#### 2.3 Chemical compositions of *S*. *psammophila* sand barriers

Samples of *S*. *psammophila* sand barriers were crushed into wood powder, then passed through a 40–60 mesh sieve and dried at 80°C to a constant weight. The content of cellulose was determined by the nitric acid-ethanol method [29]. The content of hemicellulose was determined by the hydrochloric acid-DNS reducing sugar method [30]. Klason lignin was analyzed for the sand-buried section of the barrier samples (~3 g) using the standard procedure [31]. Dried sand barrier samples were hydrolyzed with 72% sulfuric acid. After drying to a constant weight at 105 ± 3°C, the precipitated solids were determined gravimetrically as Klason lignin.

### 2.4 Illumina MiSeq sequencing and bioinformatics

The Fungal community taxonomic were profiled by Illumina amplicon sequencing. Total genomic DNA was extracted from 0.5g of each subplot sample using a Fast DNA® SPIN Kit (MP Biomedicals, Solon, USA) following the instructions. We used the primer pairs ITS1F (5’-CTTGGTCATTTAGAGGAAGTAA-3’) and ITS2R (5’-GCTGCGTTCTTCATCGATGC-3’) to amplify the entire fungal internal transcribed spacer (ITS) rDNA region. Amplification of each sample was performed in triplicate using 0.4μl of TransStart®FastPfu DNA Polymerase (TransGen AP221-02) and 0.8μl (5μM) of each primer with template DNA (10 ng/μl concentration to amplify) with a final volume of 20μl. The PCR conditions were as follows: 3 min at 95°C, followed by 35 cycles of 95°C for 30 s, 55°C for 30 s and 72°C for 45 s, with a final extension of 72°C for 10 min. The PCR product was extracted from 2% agarose gel and purified using the AxyPrep DNA Gel Extraction Kit (Axygen Biosciences, Union City, CA, USA) and quantified using Quantus™ Fluorometer (Promega, USA). Purified amplicons were pooled in equimolar ratios and paired-end sequenced (2◊300) on an Illumina MiSeq platform (Illumina, San Diego, USA) according to the standard protocols by Majorbio Bio-Pharm Technology Co. Ltd. (Shanghai, China).

Raw fungal sequences of each sample were extracted based on barcodes, quality-filtered by Trimmomatic and merged by FLASH. The 300 bp reads were truncated at any site receiving an average quality score of < 20 over a 50 bp sliding window, and the truncated reads shorter than 50 bp were discarded, as were reads containing ambiguous characters. Only overlapping sequences longer than 10 bp were assembled according to their overlapping sequence. The maximum mismatch ratio of overlap region was 0.2. Reads that could not be assembled were discarded. The sequences were clustered into operational taxonomic units (OTUs) by 97% sequence identity using UPARSE (version 7.1, http://drive5.com/uparse/). The taxonomy of the most abundant read per OTU was assigned according to the UNITE (Release 7.2 http://unite.ut.ee/index.php) reference database using the Bayesian classifier [32].

### 2.5 Statistics

One-way ANOVA was used to statistically analyze the differences between the chemical composition of *S*. *psammophila* sand barriers and the physical and chemical indexes of the surrounding soil samples in different groups, and Fisher’s least significant difference (LSD) was used for the significance test. Significance was set at P < 0.05. Residuals were inspected for normality and homoscedasticity. We used SPSS 22.0 (IBM Corp., Armonk, NY, USA) statistical analysis software for data analysis and GraphPad Prism 9.0 for graphic plotting. We used the heatmap of community composition analysis to reflect the species abundance of *S*. *psammophila* sand barriers in different setting years. Display color by logarithmic value. Show the abundance changes of different species in the sample through the color block color gradient.

According to the results of the taxonomic identification, the fungal communities heatmap, PCoA analysis and Venn diagram were drawn using R software to reflect the species composition and relative abundance at a certain taxonomic level. The alpha diversity index of the fungal community at different sequencing depths was calculated using Mothur software. Diversity within communities of fungi was compared with Shannon’s diversity index, Simpson’s diversity index and Chao 1 index. Relationships between the abundance or diversity of fungi and the decaying properties in *S*. *psammophila* sand barriers were determined by Pearson’s correlation coefficient. The Cannoco 5.0 was used to draw the redundancy analysis (RDA) diagram.

## 3 Results

### 3.1 Changes in microenvironmental factors of *S*. *psammophila* sand barriers after setting

#### 3.1.1 Soil physicochemical properties

Variations in soil physicochemical properties under different setting years of *S*. *psammophila* sand barriers are shown in Table 1. Compared with soil after sand barriers setting one year, the average increase rates of available potassium in the three-year, five-year, and seven-year of *S*. *psammophila* sand barriers were approximately 9.18%, 28.07%, and 39.80%, and significant differences were observed for different setting times. The soil around seven-year sand barriers showed the highest available nitrogen, with an average content of 3.67 mg kg^−1^, which was 1.33–1.99 times that of the other groups. Soil organic matter gradually increased after setting *S*. *psammophila* sand barriers and reached 4.15 g kg^−1^after seven years, which significantly increased compared with the soil around one-year sand barriers. In addition, the average soil moisture content of one-year and three-year sand barriers were 1.49% and 2.26%, respectively, significantly lower than that of five-year (3.25%) and seven-year (3.33%) sand barriers. The soil pH was alkaline, which ranged from 8.74 to 9.42, and did not differ among setting periods.

**Table 1:** Differences in soil physicochemical properties under different setting years of *S*. *psammophila* sand barriers.

Setting years	Available phosphorus mg/kg	Available potassium mg/kg	Available nitrogen mg/kg	Organic matter g/kg	Moisture content/%	pH	Soil porosity/%
1a	2.15±0.17a	65.33±3.05d	1.84±0.40b	1.23±0.28c	1.49±0.16c	9.11±0.26ab	31.15±0.45c
3a	1.82±0.16b	71.33±2.08c	2.18±0.17b	1.71±0.23c	2.26±0.56b	9.08±0.22ab	32.34±1.29bc
5a	1.30±0.12c	83.67±1.53b	2.76±0.67ab	3.45±0.30b	3.25±0.20a	8.74±0.20b	33.63±0.68ab
7a	1.92±0.17ab	91.33±2.08a	3.67±1.19a	4.15±0.28a	3.33±0.10a	9.42±0.11a	34.63±0.61a

Note: Values with different lowercase letters in the same column differ significantly (*P <* 0.05) as determined by the LSD test.

#### 3.1.2 Main chemical compositions

**Fig 2:**
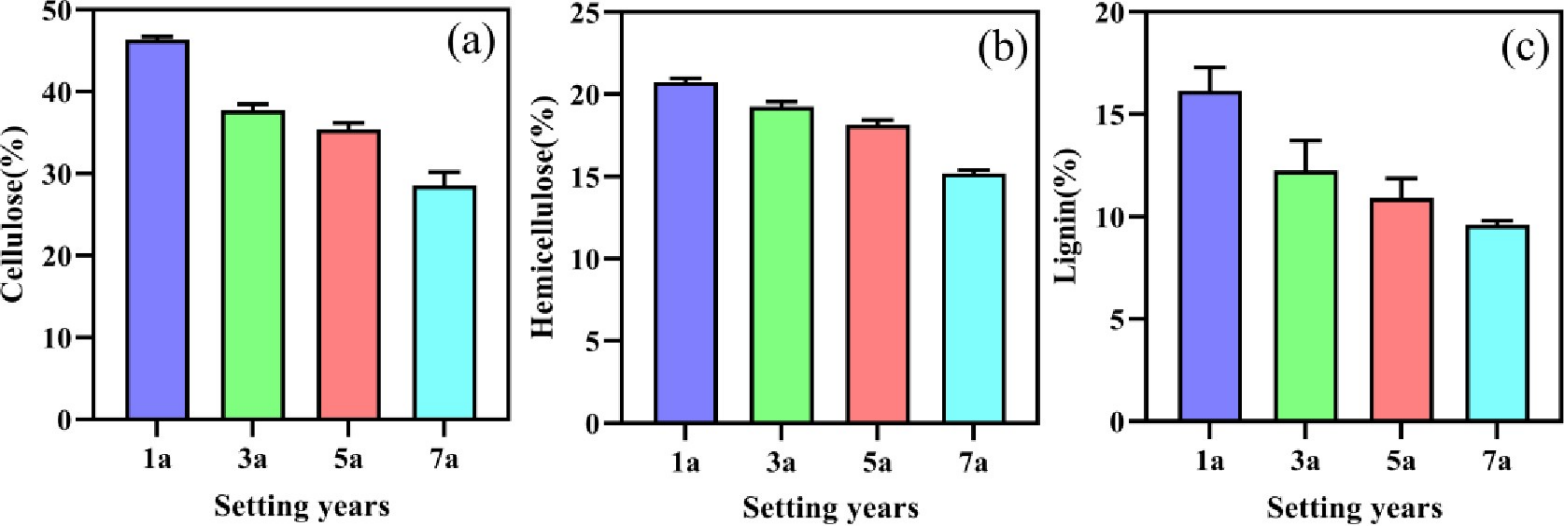
Changes in main chemical compositions under different setting years of *S*. *psammophila* sand barriers. (a): Cellulose, (b): Hemicellulose, (c): Lignin.

Samples of *S*. *psammophila* sand barriers collected from different setting years plots were analyzed, and the results for the main chemical composition of sand barriers were presented in Fig 2, such as cellulose, hemicellulose, and lignin. Our present study suggests that the chemical content of *S*. *psammophila* sand barriers decreased gradually with the increase in setting times. As shown in Fig 2A and 2B, the content of cellulose varied from 28.57% to 46.33%, while the content of hemicellulose ranged from 15.2% to 20.73%. And the amounts of these two chemical compounds differed greatly in the samples from different setting years of *S*. *psammophila* sand barriers, which could be caused by the different environment, age, and wood properties. The changes in lignin content of different periods were shown in Fig 2C. Compared with one-year sand barriers, the average decrease rates of lignin content in the three-year, five-year, and seven-year of *S*. *psammophila* sand barriers were 24.05%, 32.42%, and 40.48%, respectively, and significant differences were observed for different setting times.

### 3.2 Fungal community composition of *S*. *psammophila* sand barriers

**Fig 3:**
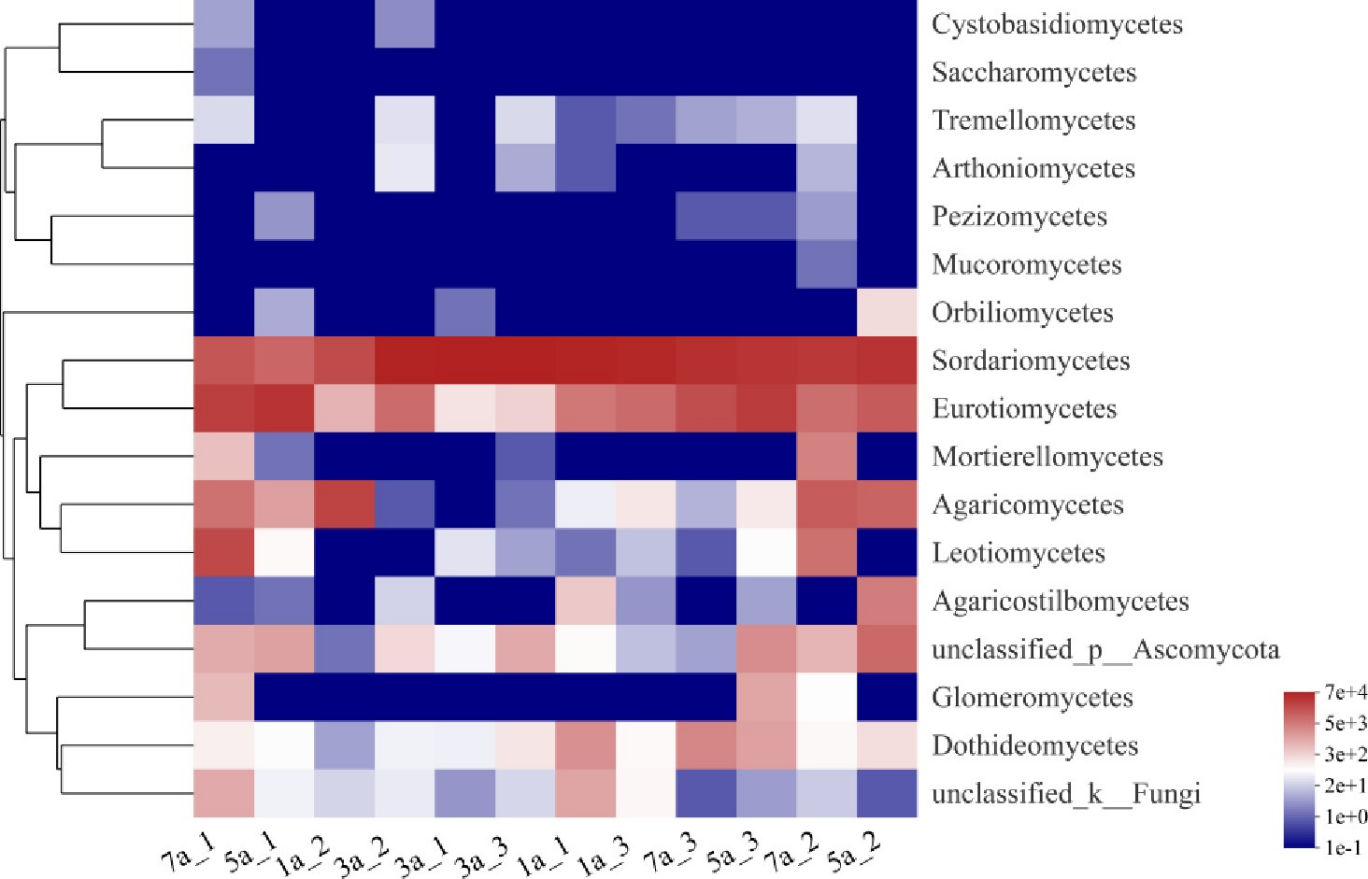
Class-level fungal communities heatmap of *S*. *psammophila* sand barriers under different setting years. The abscissa is the sample name, and the ordinate is the species name. The right side of the figure is the value represented by the color gradient, and the left side of the figure is the clustering tree of the species.

As shown in Fig 3, species abundance of Sordariomycetes was the largest in all the samples, followed by that of Eurotiomycetes. Mucoromycetes, Cystobasidiomycetes and Saccharomycetes had the lowest species abundance. The dominant and most common fungal phyla identified in all the samples were Ascomycota, followed by Basidiomycota, while Mortierellomycota and Glomeromycota were less represented (0.54% and 0.23%, respectively). Ascomycota were 90.33%, 64.12% of which Sordariomycetes, 20.11% Eurotiomycetes, and 3.29% Leotiomycetes. Nevertheless, Basidiomycota were only 8.58%, and the classes mainly represented were Agaricomycetes (8.04%), and Agaricostilbomycetes (0.52%). According to the similarity among the species, the fungi of *S*. *psammophila* sand barriers in different setting years can be clustered into two categories.

**Fig 4:**
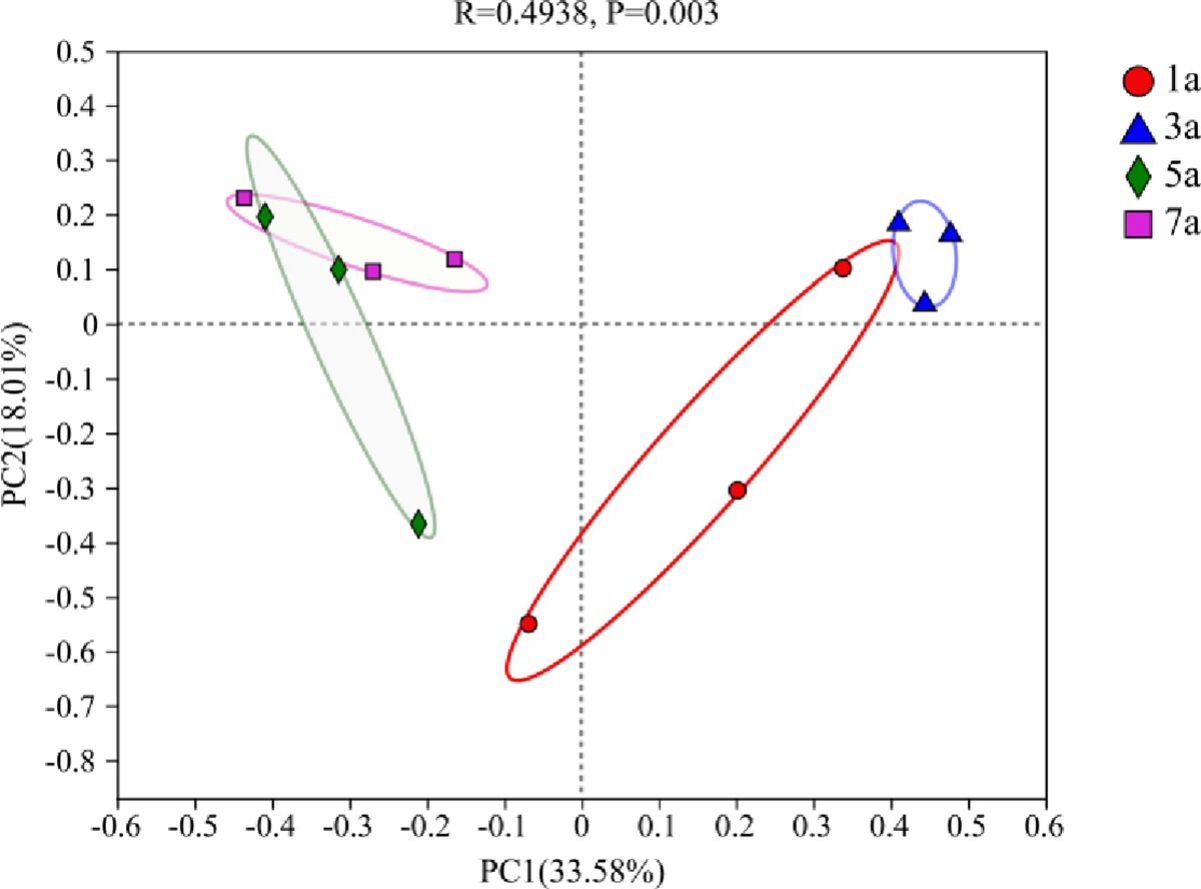
PCoA analysis of fungal communities.

Fungal communities of *S*. *psammophila* sand barriers were performed using principal coordinates analysis (PCoA) based on Bray Curtis distances. We compared the fungal community composition among all study regions. We observed an extremely significant difference in the fungal community composition of *S*. *psammophila* sand barriers with different setting years (R = 0.4938, P = 0.003, Fig 4). The fungal community composition of the five-year and seven-year *S*. *psammophila* sand barriers was relatively close. However, higher similarity in the fungal community between one-year and three-year *S*. *psammophila* sand barriers.

### 3.3 Relationships between fungal diversity and microenvironmental factors

#### 3.3.1 Richness and diversity

**Fig 5:**
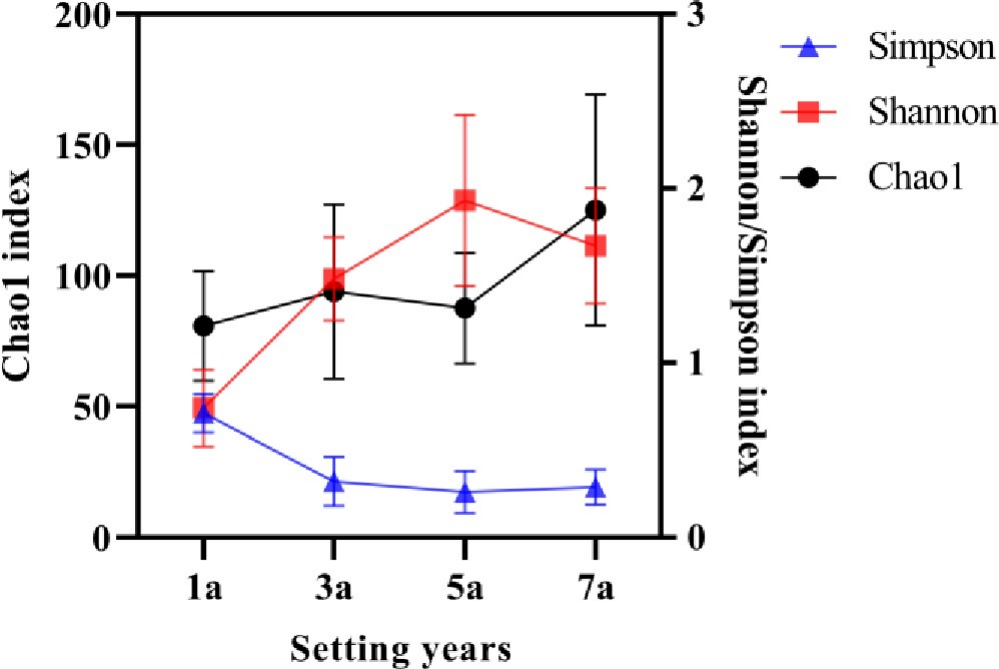
Changes of alpha diversity indices.

**Fig 6:**
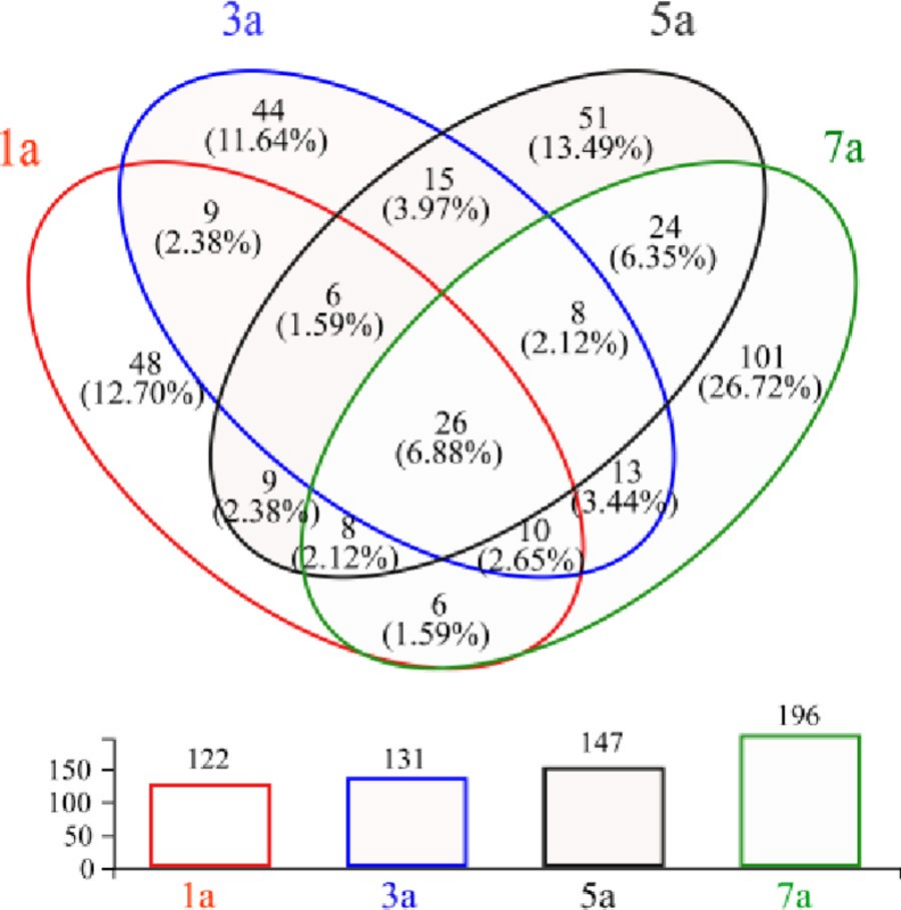
Venn diagram showing the number of OTUs.

A total of 378 fungal operational taxonomic units (OTUs, sequences based on 97% similarity) in samples of *S*. *psammophila* sand barriers that were taxonomically classified into 6 phyla, 17 class, 47 orders, 86 families, and 139 genera. At OTU level, we compared the diversity calculated by the Shannon estimator, which ranged from 0.74 to 1.93 per sample (Fig 5). Moreover, the measured richness, estimated using the Chao1 index, ranged from 80.82 to 125.06 per sample. The fungal Shannon and Chao1 index tended to gradually rise during different setting time while Simpson index had a slight decline on the whole. The alpha diversity indices demonstrated that the sample with the highest richness level was seven-year sand barriers, but the fungal diversity level was the highest in five years. Venn diagrams were calculated on specific and shared OTUs between the four different setting years of sand barriers, as shown in Fig 6, the proportions of taxa were those shared by all four sample plots: 26 (6.88%). And the number of OTUs in different samples were 122, 131, 147, and 196 respectively. Similarly, an interesting phenomenon arose in the number of specific OTUs. The high percentage of specific taxa was found in a sample of seven-year sand barriers with 101 (26.72%), while the lowest of three-year with 44 (11.64%). These results indicated that the total of fungi and specific taxa climbed with the increase in setting years.

#### 3.3.2 Correlations between microenvironmental factors and fungal alpha diversity indices

We were further interested, which microenvironmental variables influence the fungal community diversity variations most. Therefore, we performed a Pearson’s correlations analysis, which included soil physicochemical properties and main chemical composition variables. Our correlation analysis results showed that soil available potassium, soil moisture content, soil porosity, and main chemical compositions (cellulose, hemicellulose and lignin) of *S*. *psammophila* sand barriers all significantly affected the fungal diversity Simpson index (Table 2). Within the set of microenvironmental variables, particularly strong correlations with the Simpson index were found for lignin (P < 0.01). Besides, the observed variations in the fungal community diversity Shannon index were a significantly positive correlation with soil porosity (P < 0.05), while negatively correlated with lignin (P < 0.05). On the contrary, those effects were less pronounced in the Chao1 index.

**Table 2:** Correlations between microenvironmental factors and diversity index at OTU level.

Microenvironmental factors	Shannon index	Simpson index	Chao index
Available phosphorus	−0.489	0.467	0.263
Available potassium	0.570	−0.585*	0.382
Available nitrogen	0.280	−0.341	0.261
Organic matter	0.538	−0.543	0.220
Moisture content	0.550	−0.607*	0.138
pH	−0.181	0.104	0.288
Soil porosity	0.700*	−0.706*	0.543
Cellulose	−0.543	0.648*	−0.305
Hemicellulose	−0.552	0.603*	−0.420
Lignin	−0.679*	0.730**	−0.499

Note

**: Highly significant correlation at *P <* 0.01.

*: Significant correlation at *P <* 0.05.

### 3.4 Redundancy analysis

**Fig 7:**
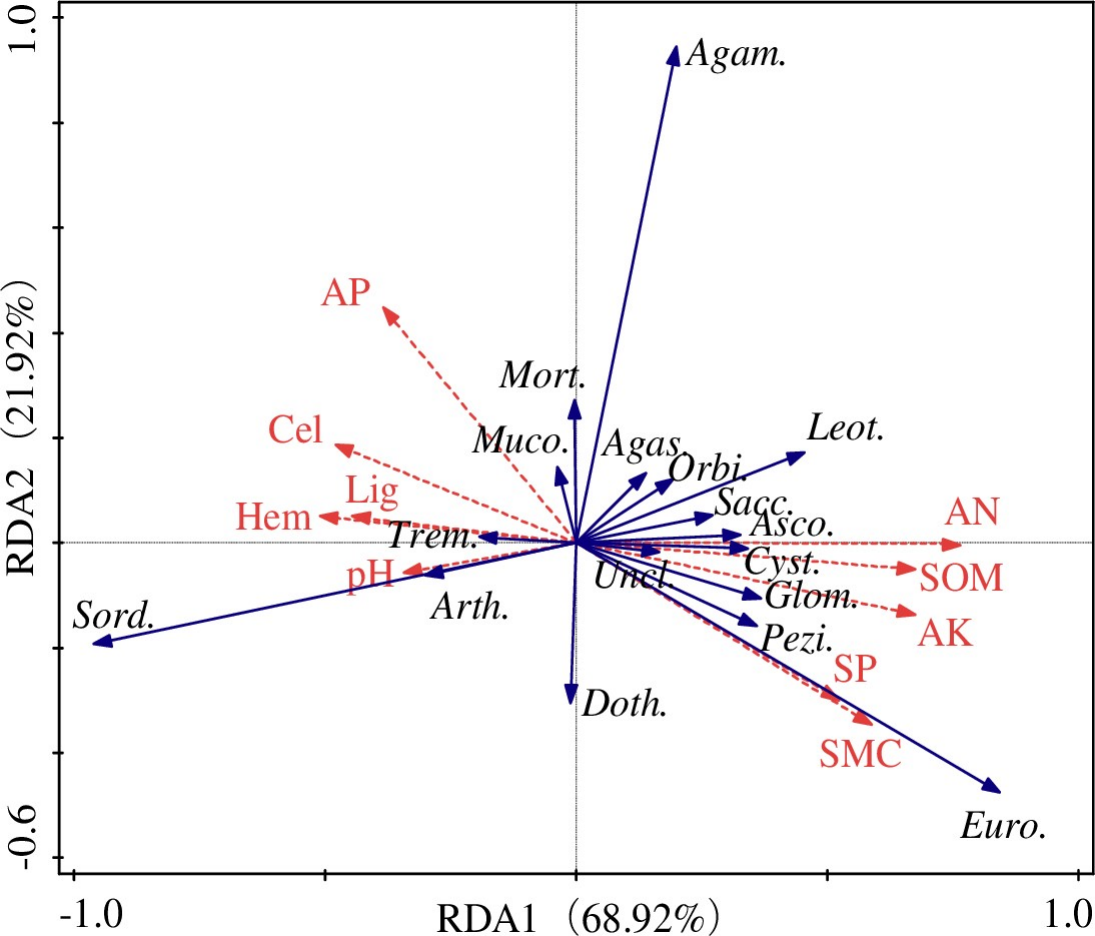
RDA analysis of microenvironmental factors and fungal communities of *S*. *psammophila* sand barriers. Cel: Cellulose; Hem: Hemicellulose; Lig: Lignin; AP: Available phosphorus; AK: Available potassium; AN: Available nitrogen; SOM: Soil organic matter; SMC: Soil moisture content; SP: Soil porosity; Sord.: Sordariomycetes; Euro.: Eurotiomycetes; Agam.: Agaricomycetes; Leot.: Leotiomycetes; Asco.: Ascomycetes; Doth.: Dothideomycetes; Mort.: Mortierellomycetes; Agas.: Agaricostilbomycetes; Uncl.: Unclassified; Glom.: Glomeromycetes; Orbi.: Orbiliomycetes; Trem.: Tremellomycetes; Arth.: Arthoniomycetes; Pezi.: Pezizomycetes; Cyst.: Cystobasidiomycetes; Sacc.: Saccharomycetes; Muco.: Mucoromycetes.

The relationship between microenvironment characteristics and the fungal community composition of *S*. *psammophila* sand barriers was analyzed by redundancy analysis (RDA). At the class level, the RDA demonstrated that fungal community compositions showed obvious correlations with microenvironment factors (Fig 7). The microenvironmental factors selected in our study could cumulatively explain 96.6% of the variation in the relationship with fungal communities. The RDA1 and RDA2 axes could explain 68.92% and 21.92% of the variation, respectively. We observed that available nitrogen, available potassium, organic matter, soil moisture content, and soil porosity were positively correlated with the RDA1 axis, while available phosphorus, soil pH, cellulose, hemicellulose, and lignin were negatively correlated with the RDA1 axis. Among the all factors, we further found that available nitrogen, available potassium, organic matter and soil moisture content had great effects on the fungal community distribution of *S*. *psammophila* sand barriers.

For fungi at the class level, the distribution of Sordariomycetes fungal communities was mainly influenced by the positive correlation between soil pH, while Eurotiomycetes was most affected by the positive correlation between soil moisture content and soil porosity. Available phosphorus had the greatest positive effect on Agaricomycetes, but nearly no effect on Agaricostilbomycetes. Otherwise, Leotiomycetes were positively correlated with soil available nitrogen and negatively correlated with soil pH.

## 4. Discussion

### 4.1 The properties of *S*. *psammophila* sand barriers and surrounding soils

Lignocellulose is the main component of *S*. *psammophila* sand barrier. With the increase in the setting period, the biological structure and chemical components have been irreversibly destroyed. Lignocellulose decomposition is reportedly rapid in the sand-buried section of the barriers [8,19]. This is also supported by our results, that is, the contents of lignin, cellulose and hemicellulose showed a decreasing trend with the increase of setting time. Among them, the lignin content of the seven-year sand barrier decreased by 40.48% compared to the one-year. Although previous studies have described the microstructure and biodegradation of sand barriers in desert ecosystems [19], our study further confirmed that the destruction of structure and components changes the physical and chemical properties of the surrounding soil. These results showed that, when the cell wall of sand barriers break down, collapse and degrade chemical components such as lignocellulose, the basic properties of the surrounding soil change as well. The ability of *S*. *psammophila* sand barriers to alter the surrounding soil of the physiochemical properties was also demonstrated by recent studies conducted in the harsh environmental conditions [6].

The decomposition of *S*. *psammophila* sand barrier in desert soil is a fundamental ecological process that is essential for nutrient cycling. Especially in nutrient-poor desert ecosystems, it appears to affect the amount of available nutrients and organic matter in the soil through wood decomposition [33]. The available nutrients can be used to evaluate soil quality, which can be directly absorbed and utilized by plant roots. In this study, we determined the contents of available nitrogen, available phosphorus and available potassium. They provide a good estimate for the ameliorating effect of *S*. *psammophila* sand barriers on desert soils. Some scholars have shown that the *S*. *psammophila* sand barriers have a significant effect on the content of soil available nutrients as the setting time increases [34]. This is consistent with the results of this study, that is, the contents of available and available nitrogen in the soil around the seven-year sand barriers were significantly higher than others. The increase of effective nutrients in the soil can promote the growth of plants. Especially in desert ecosystems where nitrogen and potassium are limited, a few available nutrient sources appear to be important for natural shrubs or herbaceous plants. In other words, *S*. *psammophila* bio-mechanical sand barriers perform several functions in desert ecosystems, including enhancing soil quality and stability, increasing the possibility of desert plants colonization.

### 4.2 Fungal community dynamics of *S*. *psammophila* sand barriers during decay

Previous studies have shown that even in extreme environments, such as dry desert areas, deterioration of wood can take place over time [35]. Although abiotic factors such as local precipitation, aeolian sand activity and temperature have been demonstrated to be crucial drivers that influence the breakdown of the lignocellulosic cell walls [8,19], biotic factors, such as the effect of microbial or fungi, also play a significant role in its degradation. According to the previous studies, the serious deterioration and decay of the sand buried section of *S*. *psammophila* sand barrier are largely caused by fungal activity in the desert environment [19]. For a more detailed comparison the decay characteristics of *S*. *psammophila* sand barrier, further studies on fungal community composition at different decay phases are necessary. We have described the composition and structural characteristics of fungal communities in the stable sand-buried of *S*. *psammophila* sand barriers with different ages, and have identified groups present in the decayed sand barriers, which could be involved in the decay and decomposition of the barriers in the arid ecosystem. To further explore the genetic functional traits of fungi, current studies have explored the potential genetic functional pathways related to lignocellulosic degradation [36]. In addition, the microbial and molecular mechanisms of lignocellulosic degradation have been investigated using meta-exo-proteome proteomics and 16S rRNA gene profiling [37]. Thus, to thoroughly assess how the dominant fungi regulate the mechanistic strategies of lignocellulose decomposition, enzymatic mechanism and potential genetic functional pathways related to lignocellulose degradation should be collectively considered and separately measured in future research.

Our results showed that Ascomycota was the dominant phylum in all samples, followed by Basidiomycota. The former mainly includes Sordariomycetes and Eurotiomycetes, while the latter mainly includes Agaricomycetes and Agaricostilbomycetes (Fig 3). These dominant phyla have also been reported in studies, such as a variety of cultivable fungi in bulk soils [38], decaying wood in dry desert areas [34], and rhizosphere microorganisms of plants in extremely arid deserts [39]. It has been reported that ninety percent of the N2O-producing fungi belong to the phylum Ascomycota, especially classes Sordariomycetes, Eurotiomycetes and Saccharomycetes [40]. There is still a large knowledge gap with regard to N2O-producing fungal species in unmanaged ecosystems such as deserts and forests [39]. However, our study confirmed that Sordariomycetes has the highest species abundance, followed by Eurotiomycetes, during a long-term decay of *S*. *psammophila* sand barrier in the desert. Saccharomycetes is the least species richness and only exists in the seven-year sand barriers. In addition, Sordariomycetes also includes many important saprophytic fungi and plant pathogens [41]. Eurotiomycetes is the most ecologically diverse fungi, which can decompose the organic residues of plants and thus play an important role in the carbon cycle of the ecosystem [42]. We also found that Agaricomycetes had a higher species abundance (8.04%). This species stands out as a plant pathogen, causing canker and white rot of the wood [43].

The RDA analysis results showed that soil available nitrogen, soil pH and soil moisture content significantly affected fungal communities of *S*. *psammophila* sand barrier (Table 3). The influence of soil available nitrogen was mainly related to decayed sand barriers and organic matter because their composition and nutrient elements enabled the survival and development of the fungal community. Additionally, the moisture content in the soil directly affects the moisture condition in the sand barrier. When there is plenty of water in the surrounding soil and the wood absorbs more water, the excellent water condition in the sand barrier can promote the growth and reproduction of fungi. The increase or decrease of moisture content will in turn affect the pH of soil or sand barrier, thus changing the growth conditions of fungi, and determining the composition and distribution of fungal community species.

**Table 3:** Importance ranking and significance test results.

Microenvironmental factors	Interpretive degree/%	Contribution/%	pseudo-F	P	Importance ranking
Available nitrogen	41.5	43.0	7.1	0.006	1
Soil pH	17.2	17.8	3.7	0.024	2
Moisture content	9.9	10.3	4.8	0.044	3
Available phosphorus	7.8	8.1	1.9	0.192	4
Cellulose	7.3	7.6	2.2	0.164	5
Organic matter	6.0	6.2	1.5	0.236	6
Hemicellulose	3.7	3.9	2.3	0.128	7
Lignin	1.5	1.5	0.7	0.558	8
Available potassium	1.2	1.2	0.6	0.626	9
Soil porosity	0.5	0.6	0.2	0.812	10

### 4.3 Relationships between fungal community diversity and microenvironmental factors

Our results suggested that the number of fungal species in the buried section of *S*. *psammophila* sand barriers tends to increase with the year of setting (Fig 6). We found that *S*. *psammophila* sand barrier has the highest fungal diversity level after five years of setting, but a slight downward trend after seven years. The main reason is that the buried section avoids the direct irradiation of ultraviolet light and easily absorbs the precipitation from the atmospheric environment or the air-condensed water formed by strong temperature differences, thus creating an excellent environment suitable for the growth of fungi [7]. Previous studies have also shown that the ability of fungi to colonize dead wood and acquire carbon compounds is enhanced by wood-soil contact that increases the water content of the wood [44,45]. The degradation of the cell wall in the *S*. *psammophila* sand barrier under long-term harsh conditions leads to a decrease in the basic density and an increase in the water absorption capacity of the sand barrier [7,19]. After seven years of setting, the lignocellulosic content and basic density of the sand barriers decreased, and it has a high capacity to absorb water. When the water is filled with the substrate, the lack of oxygen may limit the respiration of some organisms, suggesting that out of an optimal moisture range mycelial growth is inhibited [46]. Therefore, the fungal diversity of *S*. *psammophila* sand barrier showed a decreasing trend.

The associations between the fungal community diversity and microenvironmental conditions suggest that soil porosity and lignin content were significant factors affecting fungal community diversity. The increase of soil porosity can improve the oxygen flowability, which leads to the increase of the species of aerobic fungi in the *S*. *psammophila* sand barrier. Lignin is formed by three monomers of p-hydroxyphenyl propane, guaiacyl propane and syringyl propane through dehydrogenation polymerization and a disordered combination of C-C bond and C-O bond [47]. Different fungi can secrete different enzymes to degrade lignin, resulting in the breakdown of chemical bonds, the destruction of lignin structure and the decrease of lignin content. The decrease of lignin content in turn affects the species and number of fungi.

## 5. Conclusions

Our results demonstrate that the destruction of lignocellulose components during the decay process of *S*. *psammophila* sand barrier alters the physical and chemical properties of the surrounding soil. Compared with one-year sand barrier, lignin and cellulose of seven-year *S*. *psammophila* sand barrier decreased by 40.48% and 38.33%, respectively. Soil available potassium and available nitrogen increased by 39.80% and 99.46%, respectively. In a word, *S*. *psammophila* bio-mechanical sand barriers perform several functions in desert ecosystems, including enhancing soil quality and stability, increasing the possibility of desert plants colonization.

We also showed that fungal community composition is related to the decay characteristics of sand barriers and surrounding soil properties. We confirmed that soil available nitrogen, soil pH and soil moisture content significantly affected the fungal community distribution of *S*. *psammophila* sand barriers. Sordariomycetes are mainly affected by the positive correlation of soil pH, while Eurotiomycetes are most affected by the positive correlation of soil moisture content and soil porosity. It was concluded that the diversity of fungi in the sand barrier set up for five years reaches the peak value. This information may improve our understanding of the dynamic characteristics of fungal communities and decay properties during the decay process of *S*. *psammophila* sand barriers in stressful desert ecosystems.
